# Unusual external branch of the superior laryngeal nerve: a case report

**DOI:** 10.4076/1757-1626-2-7754

**Published:** 2009-06-11

**Authors:** Thejodhar Pulakunta, Bhagath Kumar Potu, Muddanna S Rao, Venkata Ramana Vollala, Soubhagya Ranjan Nayak

**Affiliations:** 1 Department of Anatomical Sciences, St. Matthew's University School of MedicineP.O. Box 30992 SMB KY1-1204, Grand Cayman, Cayman IslandsBWI; 2 Department of Anatomy, Kasturba Medical College, Manipal UniversityManipal, KarnatakaIndia; 3 Department of Anatomy, Melaka Manipal Medical College (Manipal Campus), Manipal UniversityManipal, KarnatakaIndia; 4 Department of Anatomy, Kasturba Medical College, Manipal UniversityMangalore, KarnatakaIndia

## Abstract

**Introduction:**

The knowledge of the branching pattern of external branch of the superior laryngeal nerve and its relationship with the superior thyroid artery is the key point for identifying the external branch of the superior laryngeal nerve during surgeries of the neck.

**Case presentation:**

During routine dissection of the left head and neck region of a 50 years old female cadaver, we observed a variation of the external branch of the superior laryngeal nerve around the superior thyroid artery. The external branch of the superior laryngeal nerve has presented one medial and three lateral branches. The medial branch was running on the surface of the inferior constrictor and pierced it; where as the lateral three branches are located laterally to the superior thyroid artery. The medial two lateral branches were piercing the substance of the thyroid gland, where as the lateral most branch was communicating with the left sympathetic chain.

**Conclusion:**

It is very important that surgeons carefully dissect the region of the superior pole of the thyroid gland to expose the abnormal branching pattern of external branch of the superior laryngeal nerve prior to ligation of individual thyroid vessels.

## Introduction

The external branch of the superior laryngeal nerve (EBSLN) arises with an internal branch from the superior laryngeal nerve (SLN), which is a branch of the 10th cranial nerve. The EBSLN first descends posterolaterally to the carotid arteries, crosses them, and finally passes to the larynx close to the superior thyroid artery (STA). It lies deep to these vessels. The relationship of EBSLN to the STA and the upper pole of the thyroid gland is the key point for identifying the EBSLN during surgeries of the neck. After giving off some twigs to the pharyngeal plexus and the inferior pharyngeal constrictor, the EBSLN terminates mainly within the cricothyroid muscle [1-7]. Injury to the EBSLN results in paralysis of the cricothyroid muscle and it was first described in 1906 [8]. Since then, several studies have highlighted the variations of the EBSLN in head and neck surgery [9-12,14].

## Case presentation

**Figure 1.**
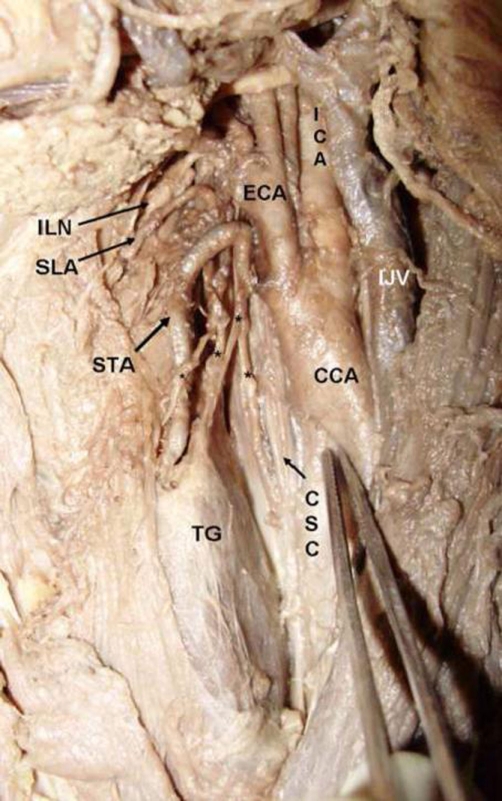
Showing the external branch of the superior laryngeal nerve and its abnormal branches. * showing the different branches of the EBSLN.

In the present case, we observed a variation in the morphological expression of the EBSLN around the STA. During routine dissection of the left head and neck region in a 50 year old female cadaver of Indian origin at the Department of Anatomy, Kasturba Medical College, we observed a variation of the EBSLN around the STA. The EBSLN has presented one medial and three lateral branches. The medial branch was running on the surface of the inferior constrictor and pierced it; where as the lateral three branches are located laterally to the STA. The medial two lateral branches were piercing the substance of the thyroid gland, where as the lateral most branch was communicating with the left sympathetic chain (Figure 1).

## Discussion

Hollinshead has described the SLN as originating from the nodose ganglion, then receiving a branch from the superior cervical sympathetic ganglion before bifurcating into the Internal laryngeal nerve (ILN) and EBSLN, implying a direct connection between the superior cervical sympathetic ganglion and the SLN itself [13]. In this case, however, we have observed communications between the cervical sympathetic chain (CSC) and the EBSLN. The incidence of CSC-ELN communication is not very common [7,14]. Many investigators have described the EBSLN as a linear structure composed of motor fiber components [2,4,6,9,12]. But in our case we found an EBSLN has different branches. These branches innervated not only the cricothyroid muscle, but also the thyroid gland.

It is opined that surgeons carefully dissect the region of the superior pole of the thyroid gland to expose the nerve trunk and its branches prior to ligation of individual thyroid vessels. The identification and exposure of nerve will be challenging when it shows different possible morphologic expressions and patterns like the one described in this case.

## Abbreviations

CSC: cervical part of the sympathetic chain
CCA: common carotid artery
ECA: external carotid artery
ICA: internal carotid artery
IJV: internal jugular vein
ILN: internal laryngeal nerve
SLA: superior laryngeal artery
STA: superior thyroid artery
TG: thyroid gland
